# (*E*)-3-Bromo-*N*′-(5-bromo-2-hydroxy­benzyl­idene)benzohydrazide

**DOI:** 10.1107/S1600536808030675

**Published:** 2008-10-04

**Authors:** Lan-Zhu Qu, Tao Yang, Guo-Biao Cao, Xiao-Ya Wang

**Affiliations:** a Department of Chemistry, Ankang University, Ankang Shanxi 725000, People’s Republic of China; b Department of Biology, Ankang University, Ankang Shanxi 725000, People’s Republic of China

## Abstract

The title compound, C_14_H_10_Br_2_N_2_O_2_, was synthesized by the reaction of 5-bromo­salicylaldehyde with an equimolar quantity of 3-bromo­benzohydrazide in methanol. The dihedral angle between the two benzene rings is 10.5 (4)°. In the crystal structure, mol­ecules are linked through inter­molecular N—H⋯O hydrogen bonds to form chains parallel to the *c* axis, and an intra­molecular O—H⋯N inter­action also occurs.

## Related literature

For related structures, see: Cao (2007*a* ▶,*b* ▶); Yang *et al.* (2008 ▶); Zhen & Han (2005 ▶); Peng & Hou (2008 ▶); Tang (2008 ▶); Salhin *et al.* (2007 ▶); Yathirajan *et al.* (2007 ▶).
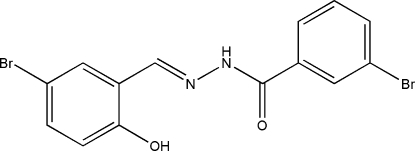


## Experimental

### 

#### Crystal data

C_14_H_10_Br_2_N_2_O_2_
*M* *_r_* = 398.06
Monoclinic, 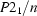

*a* = 5.657 (5) Å
*b* = 32.08 (3) Å
*c* = 7.856 (7) Å
β = 93.217 (13)°
*V* = 1423 (2) Å^3^
*Z* = 4
Mo *K*α radiation
μ = 5.70 mm^−1^
*T* = 298 (2) K
0.13 × 0.08 × 0.07 mm

#### Data collection

Bruker SMART CCD area-detector diffractometer
Absorption correction: multi-scan (*SADABS*; Bruker, 2001 ▶) *T*_min_ = 0.525, *T*_max_ = 0.691
8526 measured reflections
3227 independent reflections
1830 reflections with *I* > 2σ(*I*)
*R*_int_ = 0.044

#### Refinement

*R*[*F* ^2^ > 2σ(*F* ^2^)] = 0.048
*wR*(*F* ^2^) = 0.108
*S* = 1.06
3227 reflections
185 parameters
1 restraint
H atoms treated by a mixture of independent and constrained refinement
Δρ_max_ = 0.36 e Å^−3^
Δρ_min_ = −0.63 e Å^−3^

### 

Data collection: *SMART* (Bruker, 2007 ▶); cell refinement: *SAINT* (Bruker, 2007 ▶); data reduction: *SAINT*; program(s) used to solve structure: *SHELXTL* (Sheldrick, 2008 ▶); program(s) used to refine structure: *SHELXTL*; molecular graphics: *SHELXTL*; software used to prepare material for publication: *SHELXTL*.

## Supplementary Material

Crystal structure: contains datablocks global, I. DOI: 10.1107/S1600536808030675/bx2182sup1.cif

Structure factors: contains datablocks I. DOI: 10.1107/S1600536808030675/bx2182Isup2.hkl

Additional supplementary materials:  crystallographic information; 3D view; checkCIF report

## Figures and Tables

**Table 1:** Hydrogen-bond geometry (Å, °)

*D*—H⋯*A*	*D*—H	H⋯*A*	*D*⋯*A*	*D*—H⋯*A*
O1—H1⋯N1	0.82	1.92	2.638 (5)	145
N2—H2⋯O2^i^	0.90 (4)	1.98 (2)	2.838 (5)	160 (5)

Symmetry code: (i) 
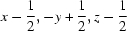
.

**Fig. 1.**
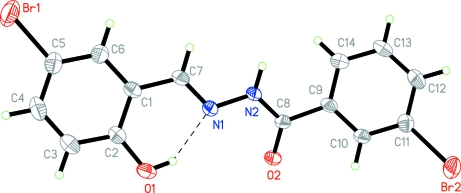
The molecular structure of (I) with ellipsoids drawn at the 30% probability level. Dashed lines indicate intramolecular hydrogen bond.

**Fig. 2.**
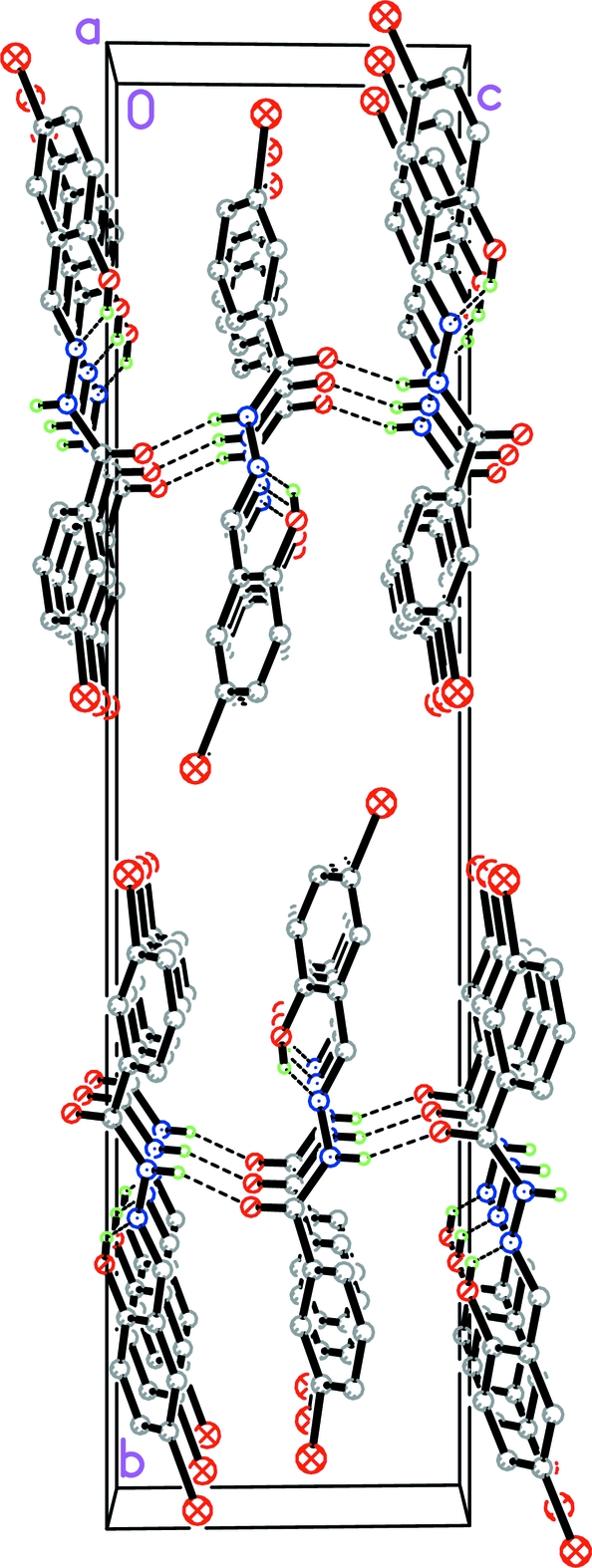
The molecular packing of (I), viewed along the a axis. Hydrogen bonds are drawn as dashed lines.

## supplementary crystallographic information

### Comment

We have recently reported some transition metal complexes with Schiff base ligands (Cao, 2007*a*,*b*) and a hydrazone compound (Yang *et al.*, 2008). We report herein the crystal structure of the title compound, (I), derived from the reaction of 5-bromosalicylaldehyde with an equimolar quantity of 3-bromobenzohydrazide in methanol.

In compound (I), Fig. 1, the dihedral angle between the two benzene rings is 10.5 (4)°. All the bond values are comparable to those in other similar hydrazones (Zhen & Han, 2005; Peng & Hou, 2008; Tang, 2008; Salhin *et al.*, 2007; Yathirajan *et al.*, 2007). In the crystal structure, molecules are linked through intermolecular N—H···O hydrogen bonds, Table 1, to form chains parallel to the c axis, Fig. 2.

### Experimental

The compound was prepared by refluxing equimolar quantities of 5-bromosalicylaldehyde with 3-bromobenzohydrazide in methanol. Colorless block-like crystals were formed when the solution was evaporated in air for about a week.

### Refinement

H2 was located in a difference Fourier map and refined isotropically, with the N–H distance restrained to 0.90 (4) Å. The other H atoms were placed in idealized positions and constrained to ride on their parent atoms, with C–H distances of 0.93 Å, the O–H distance of 0.82 Å, and with *U*_iso_(H) set at 1.2*U*_eq_(C) and 1.5*U*_eq_(O).

### Crystal data

**Table:**

C_14_H_10_Br_2_N_2_O_2_	*F*(000) = 776
*M**_r_* = 398.06	*D*_x_ = 1.858 Mg m^−^^3^
Monoclinic, *P*2_1_/*n*	Mo *K*α radiation, λ = 0.71073 Å
*a* = 5.657 (5) Å	Cell parameters from 1979 reflections
*b* = 32.08 (3) Å	θ = 2.5–25.3°
*c* = 7.856 (7) Å	µ = 5.70 mm^−^^1^
β = 93.217 (13)°	*T* = 298 K
*V* = 1423 (2) Å^3^	Block, colorless
*Z* = 4	0.13 × 0.08 × 0.07 mm

### Data collection

**Table:**

Bruker SMART CCD area-detector diffractometer	3227 independent reflections
Radiation source: fine-focus sealed tube	1830 reflections with *I* > 2σ(*I*)
graphite	*R*_int_ = 0.044
ω scans	θ_max_ = 27.5°, θ_min_ = 2.5°
Absorption correction: multi-scan (SADABS; Bruker, 2001)	*h* = −7→5
*T*_min_ = 0.525, *T*_max_ = 0.691	*k* = −40→40
8526 measured reflections	*l* = −10→8

### Refinement

**Table:**

Refinement on *F*^2^	Primary atom site location: structure-invariant direct methods
Least-squares matrix: full	Secondary atom site location: difference Fourier map
*R*[*F*^2^ > 2σ(*F*^2^)] = 0.048	Hydrogen site location: inferred from neighbouring sites
*wR*(*F*^2^) = 0.108	H atoms treated by a mixture of independent and constrained refinement
*S* = 1.06	*w* = 1/[σ^2^(*F*_o_^2^) + (0.0261*P*)^2^ + 2.1967*P*] where *P* = (*F*_o_^2^ + 2*F*_c_^2^)/3
3227 reflections	(Δ/σ)_max_ = 0.001
185 parameters	Δρ_max_ = 0.36 e Å^−^^3^
1 restraint	Δρ_min_ = −0.62 e Å^−^^3^

### Special details

**Table:**

Geometry. All esds (except the esd in the dihedral angle between two l.s. planes) are estimated using the full covariance matrix. The cell esds are taken into account individually in the estimation of esds in distances, angles and torsion angles; correlations between esds in cell parameters are only used when they are defined by crystal symmetry. An approximate (isotropic) treatment of cell esds is used for estimating esds involving l.s. planes.
Refinement. Refinement of F^2^ against ALL reflections. The weighted R-factor wR and goodness of fit S are based on F^2^, conventional R-factors R are based on F, with F set to zero for negative F^2^. The threshold expression of F^2^ > 2sigma(F^2^) is used only for calculating R-factors(gt) etc. and is not relevant to the choice of reflections for refinement. R-factors based on F^2^ are statistically about twice as large as those based on F, and R- factors based on ALL data will be even larger.

### Fractional atomic coordinates and isotropic or equivalent isotropic displacement parameters (Å^2^)

**Table:**

	*x*	*y*	*z*	*U*_iso_*/*U*_eq_	
Br1	0.52024 (14)	0.488709 (18)	0.25097 (9)	0.0871 (3)	
Br2	0.48529 (11)	0.059801 (15)	0.43970 (8)	0.0683 (2)	
O1	0.9846 (6)	0.33095 (11)	0.5203 (5)	0.0569 (9)	
H1	0.8927	0.3112	0.5117	0.085*	
O2	0.6434 (6)	0.22201 (10)	0.6015 (4)	0.0607 (10)	
N1	0.5896 (7)	0.29247 (11)	0.4185 (5)	0.0451 (9)	
N2	0.4480 (7)	0.25766 (12)	0.3888 (5)	0.0482 (10)	
C1	0.6522 (8)	0.36456 (14)	0.3700 (5)	0.0398 (10)	
C2	0.8762 (8)	0.36571 (15)	0.4560 (6)	0.0455 (11)	
C3	0.9919 (10)	0.40362 (17)	0.4777 (7)	0.0596 (14)	
H3	1.1409	0.4045	0.5339	0.072*	
C4	0.8888 (11)	0.43984 (17)	0.4170 (7)	0.0655 (15)	
H4	0.9687	0.4650	0.4317	0.079*	
C5	0.6659 (10)	0.43900 (15)	0.3338 (6)	0.0572 (13)	
C6	0.5510 (9)	0.40199 (14)	0.3089 (6)	0.0485 (12)	
H6	0.4034	0.4015	0.2505	0.058*	
C7	0.5168 (8)	0.32626 (14)	0.3474 (6)	0.0437 (11)	
H7	0.3758	0.3264	0.2806	0.052*	
C8	0.4901 (8)	0.22345 (14)	0.4857 (6)	0.0431 (11)	
C9	0.3421 (8)	0.18595 (14)	0.4356 (5)	0.0398 (10)	
C10	0.4437 (8)	0.14749 (13)	0.4676 (5)	0.0419 (11)	
H10	0.5908	0.1456	0.5261	0.050*	
C11	0.3267 (9)	0.11187 (14)	0.4127 (6)	0.0472 (12)	
C12	0.1049 (9)	0.11408 (17)	0.3310 (6)	0.0568 (14)	
H12	0.0270	0.0899	0.2939	0.068*	
C13	0.0001 (9)	0.15246 (17)	0.3051 (6)	0.0547 (13)	
H13	−0.1513	0.1542	0.2530	0.066*	
C14	0.1181 (8)	0.18839 (15)	0.3557 (6)	0.0471 (12)	
H14	0.0472	0.2142	0.3362	0.057*	
H2	0.339 (7)	0.2580 (16)	0.301 (5)	0.080*	

### Atomic displacement parameters (Å^2^)

**Table:**

	*U*^11^	*U*^22^	*U*^33^	*U*^12^	*U*^13^	*U*^23^
Br1	0.1139 (6)	0.0458 (3)	0.1014 (5)	0.0013 (3)	0.0025 (4)	0.0130 (3)
Br2	0.0836 (4)	0.0420 (3)	0.0786 (4)	−0.0039 (3)	−0.0013 (3)	0.0049 (3)
O1	0.038 (2)	0.064 (2)	0.067 (2)	−0.0044 (17)	−0.0141 (17)	0.0082 (19)
O2	0.068 (2)	0.0479 (19)	0.062 (2)	0.0009 (17)	−0.0422 (19)	0.0007 (16)
N1	0.049 (2)	0.039 (2)	0.045 (2)	−0.0038 (18)	−0.0151 (18)	−0.0009 (17)
N2	0.051 (3)	0.040 (2)	0.051 (2)	−0.0048 (19)	−0.027 (2)	0.0026 (18)
C1	0.034 (3)	0.050 (3)	0.035 (2)	−0.002 (2)	0.002 (2)	−0.006 (2)
C2	0.040 (3)	0.052 (3)	0.044 (3)	−0.007 (2)	0.001 (2)	−0.001 (2)
C3	0.050 (3)	0.069 (4)	0.058 (3)	−0.013 (3)	−0.007 (3)	−0.010 (3)
C4	0.073 (4)	0.057 (3)	0.065 (4)	−0.027 (3)	0.001 (3)	−0.006 (3)
C5	0.070 (4)	0.047 (3)	0.055 (3)	−0.007 (3)	0.006 (3)	0.002 (2)
C6	0.060 (3)	0.046 (3)	0.039 (3)	−0.002 (2)	−0.002 (2)	−0.001 (2)
C7	0.039 (3)	0.052 (3)	0.039 (3)	−0.003 (2)	−0.009 (2)	−0.002 (2)
C8	0.043 (3)	0.043 (3)	0.042 (3)	0.004 (2)	−0.011 (2)	−0.001 (2)
C9	0.042 (3)	0.049 (3)	0.027 (2)	−0.002 (2)	−0.009 (2)	0.0001 (19)
C10	0.039 (3)	0.049 (3)	0.037 (3)	0.000 (2)	−0.007 (2)	0.001 (2)
C11	0.057 (3)	0.046 (3)	0.039 (3)	−0.005 (2)	0.003 (2)	−0.001 (2)
C12	0.053 (3)	0.061 (3)	0.056 (3)	−0.022 (3)	−0.002 (3)	−0.002 (3)
C13	0.039 (3)	0.072 (4)	0.052 (3)	−0.011 (3)	−0.008 (2)	−0.004 (3)
C14	0.038 (3)	0.059 (3)	0.043 (3)	0.000 (2)	−0.001 (2)	0.001 (2)

### Geometric parameters (Å, °)

**Table:**

Br1—C5	1.893 (5)	C4—H4	0.9300
Br2—C11	1.902 (5)	C5—C6	1.362 (6)
O1—C2	1.356 (5)	C6—H6	0.9300
O1—H1	0.8200	C7—H7	0.9300
O2—C8	1.222 (5)	C8—C9	1.505 (6)
N1—C7	1.277 (5)	C9—C10	1.378 (6)
N1—N2	1.386 (5)	C9—C14	1.385 (6)
N2—C8	1.349 (6)	C10—C11	1.377 (6)
N2—H2	0.90 (4)	C10—H10	0.9300
C1—C2	1.402 (6)	C11—C12	1.379 (7)
C1—C6	1.403 (6)	C12—C13	1.376 (7)
C1—C7	1.453 (6)	C12—H12	0.9300
C2—C3	1.387 (6)	C13—C14	1.379 (6)
C3—C4	1.373 (7)	C13—H13	0.9300
C3—H3	0.9300	C14—H14	0.9300
C4—C5	1.387 (7)		
			
C2—O1—H1	109.5	N1—C7—H7	119.7
C7—N1—N2	116.2 (4)	C1—C7—H7	119.7
C8—N2—N1	118.6 (3)	O2—C8—N2	123.1 (4)
C8—N2—H2	122 (4)	O2—C8—C9	121.7 (4)
N1—N2—H2	119 (4)	N2—C8—C9	115.1 (4)
C2—C1—C6	118.9 (4)	C10—C9—C14	119.7 (4)
C2—C1—C7	122.5 (4)	C10—C9—C8	116.6 (4)
C6—C1—C7	118.6 (4)	C14—C9—C8	123.7 (4)
O1—C2—C3	118.3 (4)	C11—C10—C9	119.8 (4)
O1—C2—C1	122.3 (4)	C11—C10—H10	120.1
C3—C2—C1	119.3 (5)	C9—C10—H10	120.1
C4—C3—C2	120.7 (5)	C10—C11—C12	120.8 (5)
C4—C3—H3	119.6	C10—C11—Br2	118.6 (4)
C2—C3—H3	119.6	C12—C11—Br2	120.6 (4)
C3—C4—C5	120.3 (5)	C13—C12—C11	119.2 (5)
C3—C4—H4	119.9	C13—C12—H12	120.4
C5—C4—H4	119.9	C11—C12—H12	120.4
C6—C5—C4	119.9 (5)	C12—C13—C14	120.5 (5)
C6—C5—Br1	119.3 (4)	C12—C13—H13	119.8
C4—C5—Br1	120.8 (4)	C14—C13—H13	119.8
C5—C6—C1	120.9 (5)	C13—C14—C9	119.9 (5)
C5—C6—H6	119.5	C13—C14—H14	120.0
C1—C6—H6	119.5	C9—C14—H14	120.0
N1—C7—C1	120.6 (4)		

### Hydrogen-bond geometry (Å, °)

**Table:**

*D*—H···*A*	*D*—H	H···*A*	*D*···*A*	*D*—H···*A*
O1—H1···N1	0.82	1.92	2.638 (5)	145
N2—H2···O2^i^	0.90 (4)	1.98 (2)	2.838 (5)	160 (5)

Symmetry codes: (i) *x*−1/2, −*y*+1/2, *z*−1/2.
